# Prevalence of chronic low back pain and its associated factors in the general population of South Korea: a cross-sectional study using the National Health and Nutrition Examination Surveys

**DOI:** 10.1186/s13018-023-03509-x

**Published:** 2023-01-11

**Authors:** Hyun-Jin Park, Jun-Young Choi, Woo Myung Lee, Sang-Min Park

**Affiliations:** 1 Department of Orthopedic Surgery, Spine Center, Kangnam Sacred Heart Hospital, Hallym University College of Medicine, Seoul, Korea; 2 Spine Center and Department of Orthopedic Surgery, Seoul National University College of Medicine and Seoul National University Bundang Hospital, 82, Gumi-ro 173 Beon-gil, Bundang-gu, Seongnam-si, Gyeonggi-do 13620 Republic of Korea

**Keywords:** General population, Chronic low back pain, KNHANES, Risk factors

## Abstract

**Background:**

Many factors associated with chronic low back pain (CLBP) have been proposed, including individual, psychosocial, and physical factors. However, these associated factors are still controversial.

**Purpose:**

(1) To determine the prevalence of CLBP and (2) to analyze factors associated with CLBP in the general population using a nationally representative sample of South Koreans.

**Study design:**

Cross-sectional study.

**Patient sample:**

Data from versions IV-1, -2, and -3 of the Korea National Health and Nutrition Examination Survey (KNHANES), which were performed in 2007, 2008, and 2009, respectively (*n* = 24,871).

**Outcome measures:**

Multiple logistic regression analysis was performed to determine the association between several factors (age, gender, alcohol consumption, household income, education level, mid-intensity physical activity, depressive symptoms, vitamin D level, and comorbidities [stroke, ischemic heart disease, knee osteoarthritis, asthma, COPD, cancer history]) and CLBP.

**Methods:**

CLBP status was surveyed using a self-reported questionnaire. Demographic, socioeconomic status, comorbidities, and other factors were evaluated from health questionnaires, health and physical examinations, and laboratory tests. To analyze the association between these factors and CLBP, we used multiple logistic regression analysis.

**Results:**

Data from 17,038 participants were included in the final analysis, including 2,693 with CLBP and 14,345 without. The prevalence of CLBP was 15.8% in South Korean subjects, with a prevalence of 11.8% in men and 24.5% in women. After regression analysis, we found advanced age, female gender, mid-intensity physical activity, depressive symptoms, stroke, ischemic heart disease, knee arthritis, asthma, COPD, and cancer history were positively associated with CLBP. In contrast, alcohol consumption ≥ 1 drink per month, increased household income, higher education level, and vitamin D insufficiency were negatively associated with CLBP.

**Conclusions:**

Our study showed that CLBP was most common in the elderly and women in the general South Korean population. Several individual, socioeconomic, lifestyle, and health-related factors were associated with CLBP. These results demonstrate the influence of these factors on CLBP in the general population and suggest that consideration of these factors may improve the management of CLBP.

## Background

Low back pain (LBP), one of the most common sources of pain from musculoskeletal disorders and a major public health problem that influences the functional status and quality of life in elderly people, is reported in 7–80% of the general population at least once in their lifetime [1–4]. Patients with LBP may recover spontaneously, but some LBP patients will develop chronic LBP (CLBP) [5]. From the third decade of life until age 60, the prevalence increases linearly, with a greater frequency among women [2, 6]. In addition, a recent meta-analysis revealed that 21–68% of patients aged 60 or older had LBP in the preceding year, indicating the significant prevalence of CLBP among older persons [7]. Since the global population of people aged 60 or older is projected to quadruple by 2050 (reaching 2.1 billion), it is essential to identify risk factors for CLBP in older adults in order to design and implement appropriate preventive and treatment methods for those at high risk [8].

The risk factors for LBP are diverse, complicated, and poorly understood [9–15]. The prognosis of LBP is significantly influenced by factors unrelated to the spine. The biopsychosocial model outlines how psychological and social factors influence an individual's symptom perception [14]. Overemphasis on pain alone and reliance on a solely mechanical nominal diagnosis may lead to a worsening of disability. When treating patients with LBP, clinicians should thus address all aspects (biomechanical, psychological, and psychosocial) of the illness [16–20]. Greater pain intensity, increased body weight, carrying heavy loads at work, awkward working postures, and depression are the most frequently identified risk factors for CLBP [6]. In addition, the direct predictors of chronicity were maladaptive behavior patterns, general anxiety, functional limitation during the episode, smoking, and physical work in particular. Various biomechanical, psychological, and psychosocial prognostic factors are significant for the chronicity of LBP, according to a comprehensive investigation. In these investigations, there was a stronger correlation between CLBP and demographic, psychological, and occupational factors than with the disorder's medical features [15, 21–25]. However, these studies only evaluated the prognostic factors of patients who developed CLBP from patients with nonspecific LBP in hospital or workplace settings. In a community-based setting, no study has concomitantly examined the association between several individual, psychosocial, and physical factors, such as age, sex, alcohol intake, income, physical activities, and comorbidities, and CLBP among the general population of South Korea.

Based on this background, the aims of this study were: (1) to determine the prevalence of CLBP and (2) analyze factors associated with CLBP in the general population using a nationally representative sample of South Koreans. These results will help identify the individual risk factors for CLBP and create a reference for future efforts to decrease CLBP among the general population.

## Materials and methods

### Study participants and design

**Fig. 1:**
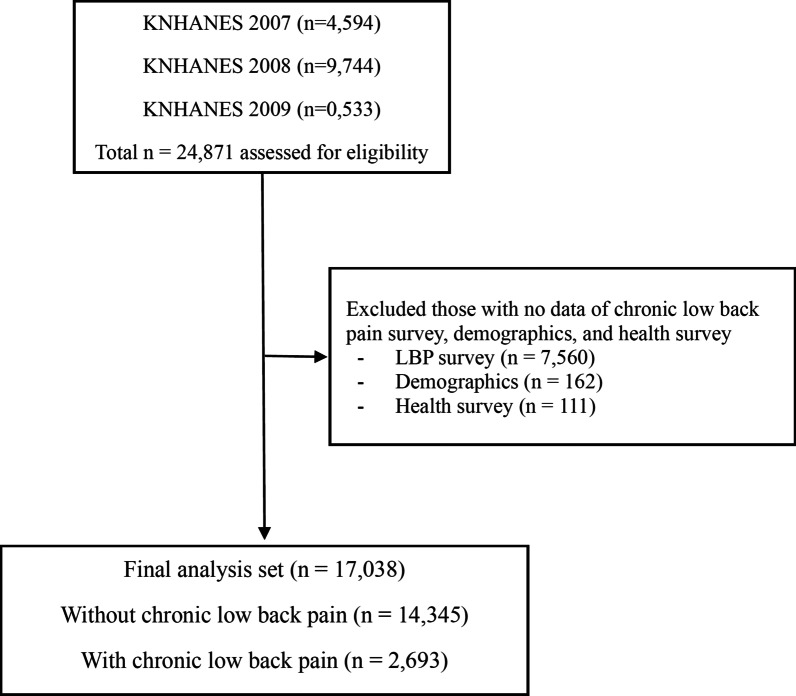
Flow diagram of the inclusion and exclusion of participants from the 2007, 2008, and 2009 Korea National Health and Nutrition Examination Surveys (KNHANES IV-1, IV-2, and IV-3, respectively). LBP, low back pain

Data from versions IV-1, -2, and -3 of the Korea National Health and Nutrition Examination Survey (KNHANES) performed in 2007, 2008, and 2009, respectively, were analyzed. This survey has been conducted annually since 1998 by the Korea Centers for Disease Control (KCDC). To evaluate the health and nutritional status of the general South Korean population, a nationwide sampling method (clustered, multistage, stratified, and randomized) is used for proportional distribution according to geographic area, sex, and age. The survey participants are different every year and are not serially monitored, resulting in a random sampling every year. The KNHANES evaluates data from three sources: health questionnaires, health and physical examinations, and nutrition questionnaires that are administered by experienced interviewers, registered nurses, and laboratory technicians [26]. The KNHANES IV-1 (2007), IV-2 (2008), and IV-3 (2009) examinations and health surveys were completed by 4,594, 9,744, and 10,533 participants (total: 24,871 participants), respectively. The present analysis was confined to 17,038 respondents aged 10–100 years who answered the CLBP examination survey and had no missing data regarding the demographics and health questionnaires (Fig. 1).

### Definition of chronic low back pain

Participants in the survey who answered “yes” to all three of the following questions were considered to have LBP: (1) “Have you ever had LBP?” (2) “Do you currently have LBP?” and (3) “Have you complained of LBP for more than 90 days during the past year?” [26, 27]

### Demographic and health surveys

We analyzed the participants’ demographics and socioeconomic status, comorbidities, and lifestyle habits through health interviews and examinations. All participants were asked whether they had received diagnoses of major comorbidities by physicians, such as hypertension, diabetes mellitus, dyslipidemia, ischemic heart disease (e.g., myocardial infarction, angina), stroke, liver cirrhosis, asthma, chronic obstructive pulmonary disease (COPD), arthritis, or chronic kidney disease. Major cancers, including lung, stomach, liver, colon, breast, prostate, uterine, and cervical cancers, were surveyed in all participants.

Age was divided into age groups. Body mass index (BMI) was calculated as bodyweight (kilograms) divided by height (meters) squared and categorized as underweight (< 18.5 kg/m^2^), normal weight (18.5–24.9 kg/m^2^), and obese (≥ 25.0 kg/m^2^) [28]. Patients were categorized into non/ex-smokers or current smokers based on their present smoking status. Alcohol consumption was categorized as none or ≥ 1 drink/month. Occupations were divided into five groups: unemployed (e.g., students, housewives), office workers (e.g., managers, professionals), sales and services, machine fitting and simple labor (e.g., technicians, device and machine operators, low-level laborers), and agriculture, forestry and fishery [29]. Household income was divided into four quartiles. Education level was categorized into four groups according to the highest level of education completed: elementary school (≤ 6 years), middle school (7–9 years), high school (10–12 years), and university or college (≥ 13 years). Physical activity was divided into three categories: “walking” was defined as walking for ≥ 30 min, 5 or more days per week; “moderate physical activity” was defined as mid-intensity physical activity for ≥ 30 min, 5 or more days per week; and “high physical activity” was defined as high-intensity physical activity for ≥ 20 min, 3 or more days per week [12]. Participants in this survey who felt sad or had a depressive symptom for 2 consecutive weeks during the past year were considered to have “depressive symptoms” [10]. Serum vitamin D levels were categorized into two groups: insufficiency (< 20 ng/ml) and normal (≥ 20 ng/ml).

### Statistical analysis

General demographics and co-variables were compared between the participants with and without CLBP. Student’s t-test was used to compare continuous variables, and the chi-square test was used for categorical variables. Univariate logistic regression analysis was used to identify individual factors associated with CLBP. Co-variables with a *p* value < 0.05 in the univariate analysis were subsequently evaluated by multiple logistic regression analysis using backward stepwise selection with a 0.05 significance level. Categorical variables were expressed as numbers and percentages, whereas continuous variables were expressed as means and standard deviations. Statistical significance was denoted by a two-tailed *p* value < 0.05. Odds ratios (ORs) with corresponding 95% confidence interval (CIs) were calculated accordingly. Sampling weights were applied to the study population to represent the South Korean population without bias. Statistics were performed using Stata/MP 15.1 (StataCorp., 2017, Stata Statistical Software: Release 15; College Station, TX, USA; StataCorp LP).

### Ethics statement

The IV-1, IV-2, and IV-3 versions of the KNHANES were approved by the KCDC Institutional Review Board (approval no. 2007-02CON-04-P, 2008-04EXP-01-C, 2009-01CON-03-2C). Informed consent was obtained from all participants when the surveys were conducted.

## Results

### Participant demographics according to chronic low back pain

**Fig. 2:**
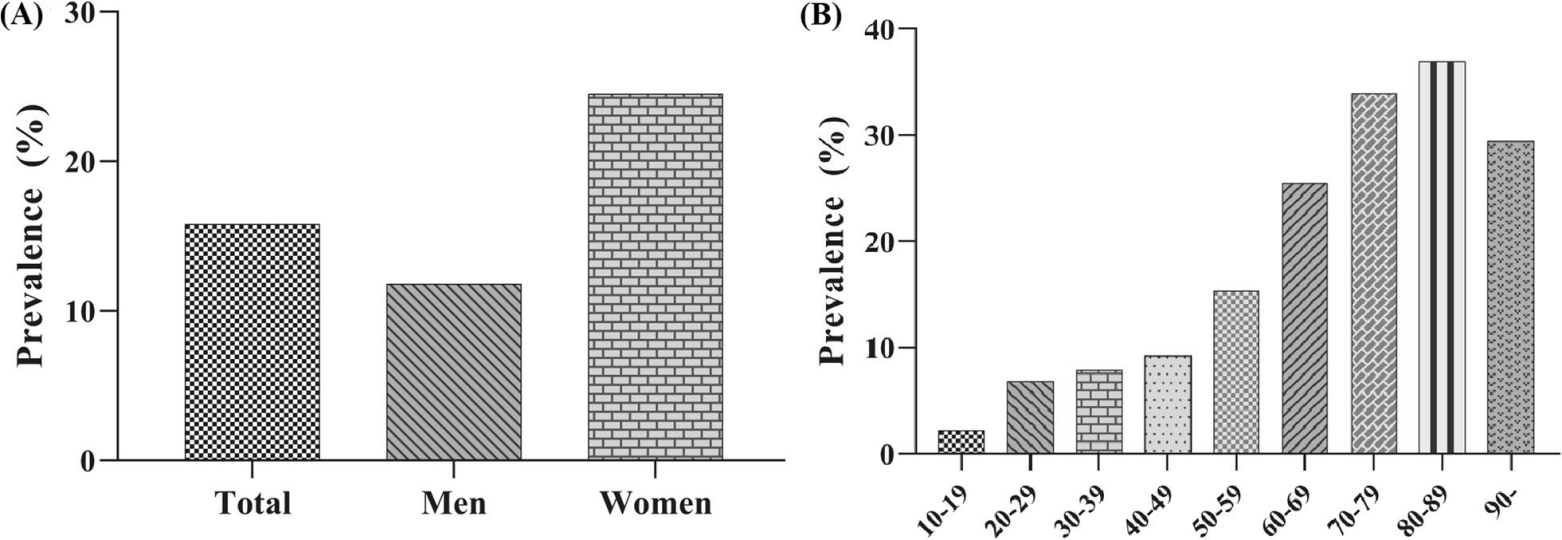
Prevalence of low back pain according to **A** gender, and **B** age group

The baseline characteristics of all participants are shown in Table 1. A total of 17,038 participants were included in the final analysis, including 2,693 with CLBP and 14,345 without. The prevalence of CLBP was 15.8% in South Korean subjects, with a prevalence of 11.8% in men and 24.5% in women. Prevalence by age group was as follows: 2.25% in teens, 6.85% in 20 s, 7.88% in 30 s, 9.22% in 40 s, 15.34% in 50 s, 25.51% in 60 s, 33.91% in 70 s, 36.93% in 80 s, and 29.41% in 90 s (Fig. 2).

**Table 1:** Characteristics of the study population according to chronic low back pain

Variables	Without CLBP (*n* = 14,345)	CLBP (*n* = 2,693)	*p* value
Age, year	47.3 (16.1)	59.1 (16.0)	< .001
Age, *n* (%)			
10–19	174 (1.2%)	4 (0.1%)	< .001
20–29	1932 (13.5%)	142 (5.3%)	
30–39	3143 (21.9%)	269 (10.0%)	
40–49	3052 (21.3%)	310 (11.5%)	
50–59	2423 (16.9%)	439 (16.3%)	
60–69	2038 (14.2%)	698 (25.9%)	
70–79	1296 (9.0%)	665 (24.7%)	
80–89	275 (1.9%)	161 (6.0%)	
≥ 90	12 (0.1%)	5 (0.2%)	
Gender, *n* (%)			
Male	6444 (44.9%)	758 (28.1%)	< .001
Female	7901 (55.1%)	1935 (71.9%)	
Height, cm	162.4 (9.1)	157.3 (9.3)	< .001
Weight, kg	62.5 (11.5)	59.0 (10.3)	< .001
BMI, kg/m^2^	23.6 (3.4)	23.8 (3.3)	.002
Obesity, *n* (%)^a^			
Underweight (< 18.5)	683 (4.8%)	106 (4.0%)	.001
Normal (18.5–24.9)	9080 (64.1%)	1644 (61.6%)	
Obese (> 25)	4403 (31.1%)	918 (34.4%)	
Smoking status, *n* (%)			
Non/Ex-smoker	11,018 (76.9%)	2304 (85.7%)	< .001
Current smoker	3318 (23.1%)	274 (14.3%)	
Alcohol consumption, *n* (%)			
None	6453 (45.0%)	1688 (62.7%)	< .001
≥ 1 drink/month	7892 (55.0%)	1005 (37.3%)	
Occupation, *n* (%)			
Unemployed (Student, housewife, etc.)	5791 (40.4%)	1375 (51.1%)	< .001
Office work	2891 (20.2%)	187 (6.9%)	
Sales and services	1893 (13.2%)	221 (8.2%)	
Agriculture, forestry and fishery	1102 (7.7%)	524 (19.5%)	
Machine fitting and simple labor	2668 (18.6%)	386 (14.3%)	
Household income, *n* (%)^b^			
Low	2572 (18.4%)	976 (36.9%)	< .001
Low-moderate	3510 (25.1%)	645 (24.4%)	
Moderate-high	3881 (27.8%)	554 (20.9%)	
High	4008 (28.7%)	471 (17.8%)	
Education level, *n* (%)^c^			
≤ 6 years	3462 (24.1%)	1503 (55.8%)	< .001
7–9 years	1594 (11.1%)	316 (11.7%)	
10–12 years	5289 (36.9%)	563 (20.9%)	
≥ 13 years	4000 (27.9%)	311 (11.5%)	
Physical activity, *n* (%)^d^			
Walk	6570 (46.0%)	1263 (47.1%)	.32
Middle PA	1903 (13.3%)	514 (19.1%)	< .001
High PA	2276 (15.9%)	408 (15.2%)	.34
Depressive symptom, *n* (%)^e^	1987 (13.9%)	698 (25.9%)	< .001
Vitamin D^f^			
Insufficiency (< 20 ng/ml)			< 0.001
Normal (≥ 20 ng/ml)	3937 (68.4%)	1059 (45.6%)	
Comorbidities, *n* (%)	6756 (63.2%)	1263 (54.4%)	
Hypertension	2591 (18.1%)	879 (32.6%)	< .001
Dyslipidemia	968 (6.7%)	305 (11.3%)	< .001
Stroke	261 (1.8%)	134 (5.0%)	< .001
Ischemic heart disease	258 (1.8%)	133 (4.9%)	< .001
Knee osteoarthritis	2119 (14.8%)	1052 (39.1%)	< .001
Asthma	512 (3.6%)	215 (8.0%)	< .001
COPD	114 (0.8%)	57 (2.1%)	< .001
Diabetes	1004 (7.0%)	310 (11.5%)	< .001
Chronic kidney disease	48 (0.3%)	17 (0.6%)	.022
Liver cirrhosis	23 (0.2%)	10 (0.4%)	.022
Cancer^g^	356 (2.5%)	145 (5.4%)	< .001

Numeric parameters are expressed as mean and standard deviation in parentheses

Categorical parameters are expressed as counts and percentages in parentheses

CLBP; chronic low back pain, BMI; body mass index, PA; physical activity, COPD; chronic obstructive pulmonary disease

^a^Body mass index was categorized into underweight (< 18.5 kg/m^2^), normal (18.5–24.9 kg/m^2^), and obese (≥ 25.0 kg/m^2^)

^b^Household income level was calculated by dividing the total household monthly income with the obtained levels then grouped into quartiles

^c^Educational level was divided into the following four groups: ≤ 6 years (elementary school), 7–9 years (middle school), 10–12 years (high school), and ≥ 13 years (college or university)

^d^Physical activity was defined as three categories. First, walk was defined as walking activity for 5 or more days per week at least 30 min. Middle physical activity was defined as mid-intensity physical activity for 5 or more days per week at least 30 min. High physical activity was defined as high-intensity physical activity for 3 or more days per week at least 20 min

^e^Depressive symptom was defined as individuals in this survey who felt sad or depressive symptom for two consecutive weeks during the past one year

^f^Serum vitamin D level were classified into two groups: Insufficiency (< 20 ng/ml) and normal (≥ 20 ng/ml)

^g^History of major cancer: stomach, liver, colon, breast, uterine cervical, prostate or lung cancer

### Unadjusted individual factors associated with chronic low back pain

Table 2 shows the results of the univariate logistic regression analysis. Underweight BMI (< 18.5), walking activity, high-intensity physical activity, and liver cirrhosis were found to be statistically insignificant.

**Table 2:** Unadjusted logistic regression analysis for all individual factors associated with chronic low back pain

Variables	Odds radio	95% CI		*p* value
Age group				
10–19	Reference			
20–29	3.197	1.169	8.741	0.024
30–39	3.723	1.371	10.110	0.01
40–49	4.418	1.629	11.987	0.004
50–59	7.881	2.910	21.346	< 0.001
60–69	14.898	5.509	40.292	< 0.001
70–79	22.321	8.248	60.406	< 0.001
80–89	25.467	9.275	69.929	< 0.001
≥ 90	18.125	4.298	76.427	< 0.001
Gender				
Male	Reference			
Female	2.082	1.902	2.279	< 0.001
Height	0.940	0.935	0.944	< 0.001
Weight	0.972	0.968	0.976	< 0.001
BMI	1.020	1.008	1.032	0.002
Obesity^a^				
Underweight (< 18.5)	Reference			
Normal (18.5–24.9)	1.167	0.944	1.441	0.153
Obese (> 25)	1.343	1.082	1.668	0.008
Smoking status				
Non/Ex-smoker	Reference			
Current smoker	0.555	0.495	0.622	< 0.001
Alcohol consumption				
None	Reference			
≥ 1 drink/month	0.487	0.447	0.530	< 0.001
Occupation				
Unemployed (Student, housewife, etc.)	Reference			
Office work	0.272	0.232	0.319	< 0.001
Sales and services	0.492	0.423	0.572	< 0.001
Agriculture, forestry and fishery	2.003	1.777	2.257	< 0.001
Machine fitting and simple labor	0.609	0.539	0.688	< 0.001
Household income^b^				
Low				
Low-moderate	0.484	0.433	0.541	< 0.001
Moderate-high	0.376	0.335	0.422	< 0.001
High	0.310	0.274	0.349	< 0.001
Education level^c^				
≤ 6 years	Reference			
7–9 years	0.457	0.399	0.523	< 0.001
10–12 years	0.245	0.221	0.273	< 0.001
≥ 13 years	0.179	0.157	0.204	< 0.001
Physical activity^d^				
Walk	1.043	0.960	1.133	0.316
Middle PA	1.542	1.385	1.717	< 0.001
High PA	0.946	0.844	1.061	0.341
Depressive symptom^e^	2.176	1.972	2.401	< 0.001
Vitamin D^f^				
Insufficiency (< 20 ng/ml)	Reference			
Normal (≥ 20 ng/ml)	0.695	0.635	0.761	< 0.001
Comorbidities				
Hypertension	2.198	2.007	2.408	< 0.001
Dyslipidemia	1.765	1.541	2.022	< 0.001
Stroke	2.826	2.285	3.495	< 0.001
Ischemic heart disease	2.837	2.292	3.512	< 0.001
Knee osteoarthritis	3.699	3.380	4.048	< 0.001
Asthma	2.344	1.988	2.764	< 0.001
COPD	2.699	1.959	3.720	< 0.001
Diabetes	1.729	1.511	1.978	< 0.001
Chronic kidney disease	1.892	1.087	3.295	< 0.001
Liver cirrhosis	2.321	1.103	4.882	0.026
Cancer^g^	2.236	1.836	2.725	< 0.001

95% CI, 95% confidence interval, BMI; body mass index, PA; physical activity, COPD; chronic obstructive pulmonary disease

^a^Body mass index was categorized into underweight (< 18.5 kg/m^2^), normal (18.5–24.9 kg/m^2^), and obese (≥ 25.0 kg/m^2^)

^b^Household income level was calculated by dividing the total household monthly income with the obtained levels then grouped into quartiles

^c^Educational level was divided into the following four groups: ≤ 6 years (elementary school), 7–9 years (middle school), 10–12 years (high school), and ≥ 13 years (college or university)

^d^Physical activity was defined as three categories. First, walk was defined as walking activity for 5 or more days per week at least 30 min. Middle physical activity was defined as mid-intensity physical activity for 5 or more days per week at least 30 min. High physical activity was defined as high-intensity physical activity for 3 or more days per week at least 20 min

^e^Depressive symptom was defined as individuals in this survey who felt sad or depressive symptom for two consecutive weeks during the past one year

^f^Serum vitamin D level were classified into two groups: Insufficiency (< 20 ng/ml) and normal (≥ 20 ng/ml)

^g^History of major cancer: stomach, liver, colon, breast, uterine cervical, prostate or lung cancer

### Association between individual factors and chronic low back pain

Table 3 shows the results from the multiple logistic regression analysis. The included associated factors were age, gender, alcohol consumption, household income, education level, mid-intensity physical activity, depressive symptoms, vitamin D level, and comorbidities (e.g., stroke, ischemic heart disease, knee osteoarthritis, asthma, COPD, and cancer history). Among these factors, advanced age, female gender, mid-intensity physical activity, depressive symptoms, stroke, ischemic heart disease, knee arthritis, asthma, COPD, and cancer history were positively associated with LBP. In contrast, alcohol consumption ≥ 1 drink per month, increased household income, higher education level, and vitamin D insufficiency were negatively associated with CLBP.

**Table 3:** Association between individual factors and chronic low back pain using multiple logistic regression

Variables	Odds radio	95% CI		*p* value
Age group				
10–19	Reference			
20–29	3.113	1.127	8.593	0.028
30–39	3.454	1.259	9.474	0.016
40–49	3.136	1.144	8.594	0.026
50–59	4.090	1.490	11.230	0.006
60–69	6.104	2.220	16.787	< 0.001
70–79	8.996	3.260	24.823	< 0.001
80–89	8.778	3.103	24.838	< 0.001
≥ 90	6.474	1.116	37.544	0.037
Gender				
Male	Reference			
Female	1.763	1.566	1.985	< 0.001
Alcohol consumption				
None	Reference			
≥ 1 drink/month	0.864	0.774	0.964	0.009
Household income^a^				
Low	reference			
Low-moderate	0.869	0.756	0.999	0.048
Moderate-high	0.906	0.779	1.054	0.200
High	0.889	0.757	1.044	0.152
Education level^b^				
≤ 6 years	reference			
7–9 years	0.741	0.628	0.874	< 0.001
10–12 years	0.604	0.514	0.710	< 0.001
≥ 13 years	0.447	0.367	0.545	< 0.001
Middle PA^c^	1.520	1.339	1.726	< 0.001
Depressive symptom^d^	1.551	1.374	1.750	< 0.001
Vitamin D^e^				
Insufficiency (< 20 ng/ml)	0.767	0.693	0.850	< 0.001
Normal (≥ 20 ng/ml)	Reference			
Comorbidities				
Stroke	1.532	1.169	2.007	0.002
Ischemic heart disease	1.590	1.207	2.094	0.001
Knee osteoarthritis	1.663	1.477	1.873	< 0.001
Asthma	1.434	1.164	1.767	0.001
COPD	2.091	1.378	3.173	0.001
Cancer^f^	1.416	1.104	1.816	0.006

OR, Odds ratio; 95% CI, 95% confidence interval, PA; physical activity, COPD; chronic obstructive pulmonary disease

^a^Household income level was calculated by dividing the total household monthly income with the obtained levels then grouped into quartiles

^b^Educational level was divided into the following four groups: ≤ 6 years (elementary school), 7–9 years (middle school), 10–12 years (high school), and ≥ 13 years (college or university)

^c^Middle physical activity was defined as mid-intensity physical activity for 5 or more days per week at least 30 min

^d^Depressive symptom was defined as individuals in this survey who felt sad or depressive symptom for two consecutive weeks during the past one year

^e^Serum vitamin D level were classified into two groups: Insufficiency (< 20 ng/ml) and normal (≥ 20 ng/ml)

^f^History of major cancer: stomach, liver, colon, breast, uterine cervical, prostate or lung cancer

## Discussion

We analyzed several factors associated with LBP in a representative sample of 17,038 South Koreans. The prevalence of CLBP was 15.8% in the general South Korean population over 10 years of age. Many individual factors were associated with CLBP in our participants. Among these, advanced age, female gender, mid-intensity physical activity, depressive symptoms, stroke, ischemic heart disease, knee arthritis, asthma, COPD, and cancer history were positively associated with CLBP. On the other hand, alcohol consumption of ≥ 1 drink per month, increased household income, increased education level, and vitamin D insufficiency were negatively associated with CLBP.

### Prevalence, age, and gender

In a review of the worldwide prevalence of LBP that included 54 countries, the mean prevalence of CLBP was estimated to be 20.6% (95% CIs 19.4–21.9%) [8]. We found that the prevalence of CLBP in the general South Korean population was 15.8% (11.8% in men and 24.5% in women), which is slightly lower than that previously reported.

Old age and female gender are well-established risk factors for CLBP [6, 8]. At 70 years of age, the OR increased to 8.996. In a previous study, the CLBP prevalence was highest in those aged 45–64 years [30]. However, in our study, the positive association between age and CLBP increased up to the 8th decade of life. The increased risks in women are similar to those in a previous study [21]; as age increases, the prevalence of CLBP increases, and advanced age is regarded as a risk factor [31, 32]. On the other hand, it has also been reported that age is not associated with CLBP or radiating to the lower extremity pain [33]. In our study, the ORs increased with increasing age, reaching a peak in the 70 s, and decreased in the 90 s. Female gender is known to be a risk factor and had an OR of approximately 1.54 in a previous study [33]. In our study, the OR was 1.763, which is similar to that previously reported.

### Lifestyle factors

In previous studies, smoking and alcohol were reportedly associated with CLBP [31–34]. In particular, smoking varies according to the study, but it has been consistently observed as a risk factor for CLBP. Alcohol consumption was found to be a significant risk factor in some studies but was not reported by a systematic review [32]. However, the results of our study suggest that smoking is not a relevant factor for CLBP, and alcohol consumption is rather negatively associated with CLBP. However, alcohol consumption cannot be considered a protective factor due to the inability of this study to determine a causal relationship. Future studies are needed.

### Socioeconomic factors

Socioeconomic factors, such as education level, household income, and occupation, are widely accepted as factors that are associated with multiple health outcomes. In previous studies, these factors were also found to be associated with CLBP [33, 35, 36]. Similar to these studies, we found that education level was strongly negatively associated with CLBP [33, 35]. Because education level is associated with an understanding of health and treatment, similar results are likely to be seen in several studies. Household income and occupation were significantly associated with LBP in the univariate analysis, but not in multivariate analysis. In our study, education level was the only socioeconomic factor consistently found to be associated with CLBP.

### Physical activity and BMI

Walking and regular physical activity is an excellent method of pain reduction for patients with CLBP [16, 37]. Previous studies have reported that walking reduces CLBP [12], and that physical activity significantly reduces LBP in those who sit for extended periods [9]. Furthermore, physical activity is especially important in patients with high BMIs [38, 39]. In this study, obesity, walking, and high-intensity physical activity were not independently associated with CLBP. In addition, mid-intensity physical activity was found to be positively associated with CLBP. Previous studies have shown that strenuous exercise is associated with CLBP [40]; however, no association has been reported between mid-intensity exercise and LBP. It is unclear due to the cross-sectional study design, and determining a causal relationship may be difficult in this circumstance because patients with CLBP can only perform mid-intensity exercises.

### Depressive symptom

Psychiatric problems, including depression, are associated with CLBP and are considered to be risk factors for increasing CLBP [41]. Among them, depression was reported to be closely associated with CLBP, radiation pain, and poor surgical outcomes and was reported relatively consistently in many papers [33, 41, 42]. In this study, the results were in line with previous studies and showed a high OR.

### Vitamin D

Numerous studies have demonstrated the association between vitamin D deficiency and CLBP [43, 44]. This association is supported by several theories, but the exact mechanism is not yet clear [43]. Furthermore, some have refuted the association between vitamin D levels and CLBP [45], and others have determined that vitamin D supplementation is not effective for controlling CLBP [46]. However, our study showed the opposite result; vitamin D deficiency was negatively associated with CLBP. Rather than interpreting this negative association as a result of our study, it is better to conclude that vitamin D is not associated with CLBP.

### Comorbidities

The association between comorbidities and CLBP is well established [19, 47]. In theory, comorbidities may affect allostatic loads and cause pain through the dysregulation of physiological mechanisms, but these mechanisms have not been precisely identified [47]. In a previous study, CLBP increased relatively consistently with the number of comorbidities, with an OR of 5.05 when there were four or more comorbidities. In this study, hypertension, dyslipidemia, diabetes, chronic kidney disease, and liver cirrhosis were not associated with CLBP, but cerebrovascular events, cardiovascular disease, pulmonary disease, knee arthritis, and cancer history were found to be positively associated with CLBP.

### Strengths and limitations

To the best of our knowledge, this is the first study investigating personal factors associated with CLBP to this extent in a representative sample of the general South Korean population. The greatest strength of our study was the increased external validity of our findings due to the KNHANES data. The KNHANES has the advantage of obtaining a large amount of data from the general population nationwide [26]. With the use of nationwide sample data, the results can be generalized to the greater community.

There are some limitations to our study. First, this study was conducted through a national health and nutrition examination survey, which was designed to be cross-sectional. Therefore, we cannot determine causal relationships between the identified associated factors and CLBP. However, as mentioned above, this dataset was extracted from the South Korean population to minimize sampling errors, and the results can be considered very representative. Second, the KNHANES was designed to minimize sampling errors by utilizing a clustered, multi-stage, random sampling method. However, selection bias may exist due to missing data. Participants were selected from our raw data to minimize selection bias, but missing data inevitably led to bias. Unlike other studies, such as cohort studies and clinical trials, the imputation of missing values is impossible in our dataset. Therefore, we excluded participants with missing data, which was necessary for analysis. Third, the simple CLBP survey used in this study did not evaluate the severity, source, or duration of LBP, which would require instruments for measuring pain on a scale (e.g., the visual analog scale pain score). Fourth, this study could not analyze some prognostic factors for CLBP, such as previous episodes of CLBP, the severity of pain, and disability. However, this study concomitantly analyzed many other associated factors that were not included in other studies. Finally, our study produced slightly different results from those of previous studies. These previous studies determined associations by analyzing relatively few factors, but our study analyzed nearly all the individual, socioeconomic, lifestyle, and mental factors and comorbidities associated with CLBP. To control for confounding factors, we used multiple logistic regression analysis to assess meaningful associations. We believe this method is an excellent statistical technique for identifying associated factors [48].

## Conclusion

Our study showed that CLBP was most common in women and the elderly in South Koreans over ten years of age in the general population. Several individual, socioeconomic, lifestyle, and health-related factors were associated with CLBP. Among these factors, advanced age, female gender, mid-intensity physical activity, depressive symptoms, stroke, ischemic heart disease, knee arthritis, asthma, COPD, and cancer histories were positively associated with CLBP. In contrast, alcohol consumption ≥ 1 drink per month, increased household income, higher education level, and vitamin D insufficiency were negatively associated with CLBP. These results demonstrate the influence of these factors on CLBP in the general population and suggest that consideration of these factors may improve the management of CLBP.

## Data Availability

The datasets used and analyzed during the current study are available from the corresponding author on reasonable request.

## Abbreviations

LBP: Low back pain
KNHANES: Korea National Health and Nutrition Examination Survey
KCDC: Korea Centers for Disease Control
COPD: Chronic obstructive pulmonary disease
BMI: Body mass index
ORs: Odds ratios
CIs: Confidence interval
